# Topics of Public Interest

**Published:** 1909-04

**Authors:** 


### TOPICS OF PUBLIC INTEREST.

### The Chemistry of Smoking

BY H. IRVING HANCOCK

*Sunday Magazine, New York Tribune, February, 28 1909.*

THE under dog was filling his pipe, at which the medical student looked on with a knowing smile. The under dog is a graduate in, or student of, chemistry, who is getting his first practical laboratory experience by serving as a chemist's helper. The medical student liked to lounge in the laboratory because he felt that the atmosphere was helpful.

"Why don't you cut that out?" inquired the medical student, nodding at the pipe.

"Why should I?" cross examined the under dog.

"Don't you know that you're filling your system with nicotine? Don't you know that nicotine is a very deadly poison?" warned the medical student. "Say, if you want to get an idea of the nicotine that's in your tobacco, light your pipe and blow some mouthfuls of the smoke through a clean white handkerchief. Then look at the big brown stains of nicotine you'll get from that smoke!"

"Do you call that brown stain nicotine?" asked the chemist, from the corner of the room.

"Yes, isn't it?" asked the medical student, growing slightly red in the face.

The trick of blowing tobacco smoke through a white handkerchief is so old that it has become classic. The truth is that as an experiment it's a fraud. The brown stain is not due to nicotine, but to the condensation of tar that has just been distilled from the woody fiber of tobacco. Nicotine itself is a nearly colorless alkaloid, and is present in smoking tobacco only in small quantities. The brownish "juice" that is found in the stems of pipes is not nicotine, but mainly a mixture of tar and water.

There are a good many fallacies current regarding tobacco and smoking. One of them relates to the quantity of nicotine that a smoker absorbs. It is so small as to be practically insignificant. In the tobacco plant at maturity there is considerable nicotine; but the leaves are put through a process of curing and sweating, and, being a very volatile substance, a large percentage of the nicotine is driven from the tobacco during the sweating.

When a pipe, a cigar, or a cigarette is lighted, the heat promptly volatilises the nicotine still remaining in the tobacco. For that reason nearly all the nicotine passes from the tobacco to the atmosphere. Some of the smoke is drawn into the smoker's mouth; but is usually promptly expelled, carrying with it nearly all traces of nicotine.

While nicotine is popularly known as the poison of tobacco, few people know anything of nicotine's chemical relatives, the pyridine bases, which also exist in tobacco. In burning, the tobacco sets them free as it does the nicotine. Up to the present, experiments in physiological laboratories have not demonstrated conclusively that appreciable quantities of either nicotine or the pyridine bases enter the system of the smoker.

This is not to be taken, however, as a statement that nicotine and its chemical cousins do no harm whatever to weed users. It is possible, probable, that almost infinitesimal quantities do enter the smoker's system, and these, by cumulative effect, may in time work harm.

### POISONS THAT DO INJURE.

There are two other poisons always to be found in tobacco smoke, one inert in small quantities, the other more active. The burning of tobacco in air forms, by combination with oxygen, two gases with which every reader is familiar. One of these is carbon dioxid, or carbonic acid; the other is carbon monoxid, the gas that is seen to burn with a blue flame at the top of a coal fire. The volume of carbon dioxid given off in tobacco smoke is large; that of its more dangerous companion, carbon monoxid, is small.

How do any of these poisons enter the system of the smoker? First of all, they are capable of being absorbed to some extent through the mucous lining of the mouth. But it is in the lungs of one who inhales that the great harm is done. This senseless practice of sucking the smoke down into the lungs keeps up a constant and dangerous irritation of the delicate, sensitive membranes of those valuable organs. The burning of paper is blamed for much of the harm that cigarettes sometimes do; but to the delicate membranes of the lungs the tar abundant in tobacco smoke is much more harmful. And the poisons present in tiny quantities in tobacco smoke pass through the linings of a lung direct to the blood.

To the inhaler smoking is therefore a practice that tends to quick physical degeneration. For the smoker who never allows the vapor to go lower than his mouth, the cigarette is probably no more harmful than the pipe or cigar. Every smoker, however, who has the "cigarette cough" is an inhaler, whether he admits it or not.

Apart from diseases of the lungs, caused by inhaling, many medical authorities charge that persistent smoking causes hardening of the arteries and angina pectoris. In discussing this charge, it may be remarked that most smokers appear to die from other causes. Just how smoking operates to cause deposits of lime along the walls of the arteries is not quite clear.

Among smokers, at least, there is a very common idea that tobacco smoke is a valuable disinfectant. It is wholly probable that, at the moment when one is smoking, the fumes will do more or less damage to bacteria just then entering the mouth. That the use of tobacco renders the system of the smoker at all immune at any time when he is not smoking is extremely improbable.

That germs cannot exist on tobacco is another common delusion. Yet one of the manufacturing methods of improving the flavor of an inferior tobacco consists in first destroying, with formaldehyde, the bacteria already growing in that tobacco. The next step consists of "sowing" on the sterilised tobacco other kinds of bacteria. This is done under certain conditions of temperature and humidity, and results in a tobacco now possessing a flavor much more agreeable to the user.

---

### An Unclean and Dangerous Habit

(*New York Tribune*, January 10, 1909.)

ACROSS the river in New Jersey a wealthy business man has had himself sworn in as special officer to enforce the law against spitting in street-cars. He has found that anti-spitting ordinances do not enforce themselves, and that a large number of men can look the sign in the front of the street car in the eye without flinching while violating an ordinance which has a good sized penalty attached to it in case of conviction.

A Brooklyn man who has occasion to use the elevated cars about midnight complains that the same carelessness is shown on this side of the Hudson River. The sign is there, warning all and sundry of the dire consequences to follow spitting, and while this unquestionably has its effect, the nuisance is by no means sufficiently abated. A few nights ago he walked through the cars in the hope of finding a seat where he might sit without violating his perhaps too esthetic sensibilities, but in vain. He was compelled to take the least obnoxious place. No car attendant has ever been known to take any interest in this matter, and among violators of the rule are frequently numbered the policemen who ought to be expected to assist in the enforcement of law.

An exhibit is now being conducted in this city with the definite purpose of disseminating information as to the methods of preventing the spread of tuberculosis. At that exhibit particular attention is paid to the danger from careless spitting. In the street cars we have signs put up by a local anti-tuberculosis society advising all who ride in cars not to communicate tuberculosis to others or permit them to be the agents of spreading the disease through this means. Yet men who would be insulted if assured that they were not gentlemen are guilty of this practice, which is not only unnecessary and disgusting but fraught with grave danger to the community. A few public spirited men like the one who has taken up the fight for health and cleanliness in New Jersey would add much to the satisfaction of the women and well bred men who ride in our cars.

---

### The State Charities Aid Association of New York

This association has issued the following bulletin:

WHY SPIT ON THE FLOOR?

*More arrests should be made.*

NEW YORK, March 15, 1909. "$500.00 fine or one year in prison, or both." This is part of the following sign appearing in the New York subway trains and surface cars, daily read by thousands of people. "By Order of the Board of Health. Spitting on the floor of this car is a misdemeanor $500 fine, or imprisonment for one year, or both, may be the punishment therefor.—Sec. 194 Sanitary Code, Sec. 151 Penal." Whether it is due to the fear of the penalty, or the general enlightment of the public on the subject, is uncertain, but the fact remains that the spitting evil has largely diminished in New York in the past decade, although from time to time drastic measures are employed by the Department of Health to further impress this law on the mind of the careless public. Only a few days ago Commissioner Darlington called out a special squad of policemen and had them on duty at some of the subway stations in this city. One hundred and eighty-four arrests were made, 681 having been made since January 1, 1909. Of course, the effect on the individual arrested is the least of the benefit to be expected from this rational enforcement of the law. It should leave a lasting expression on those who witness the arrests and those who read of them.

There is too great an apathy in regard to certain laws and ordinances on the statute books of most cities in this state: they are simply dead letters. The investigations conducted by the State Charities Aid Association in connection with the campaign for the prevention of tuberculosis have revealed that nearly every city has a spitting ordinance, but in the majority of them it is not enforced and arrests have not been made. The attitude of the average American citizen when he reads a sign, "Don't Spit on the Floor. Penalty $500.00" is that this injunction is an enfringement on his inalienable right as an American citizen to spit when and where he pleases. Doubtless, if the sign should read so as to appeal to his reason it might have a different effect. In some cities the sign, "Why Spit on the Floor? It Spreads Disease," is used. And while each person spitting doubtless feels that the sign is not meant for him, he is suspicious of his neighbor who indulges in the filthy practice, and this impression reacts as a check on his own carelessness. Nevertheless, there is an inexcusable amount of spitting done in every city.

Now, that the danger of this evil is known, not only to those few who are interested professionally or otherwise, but to the public as well, there is no excuse for the condition of the public buildings and streets in some of our cities. It is time that police departments coöperated with the health department in enforcing these ordinances.

---

### Vanderbilt Tenements

**Hygienic Homes for Consumptives—$1,000,000 to go into a new System that may revolutionize New York—Is a buisness move—Has also its philanthropic side, but is intended to be self-supporting.**

[*New York Sun* special to the *Buffalo Express*, February 24, 1909.]

MRS. WILLIAM K. VANDERBILT has embarked upon a model tenement enterprise here in New York, quite different from any of the other improved tenement-building schemes with which the city in recent years has been made familiar. Mr. Vanderbilt shares his wife's interest in the new project and work has already been begun toward its realisation.

Briefly stated, the enterprise is the establishment in the form of tenement houses of a healthful home for city sufferers from tuberculosis. It represents an investment of $1,000,000 and the contractors who have already begun the work of clearing the land for the new building do not know as yet the name of their employer. In fact, they have been a little dubious about their job, in view of its magnitude, and have made uneasy inquiries of the architect who engaged them as to the financial responsibility of their real patron, whose name until today the architect has not been at liberty to make known.

The enterprise, while in its initial form, having for an object the supplying of hygienic homes for tuberculosis victims, has a larger and more important aspect, for it comprehends the opening of the way for a new housing system. While there is distinctly an element of the philanthropic in its initiation, it also represents a business proposition which the prime movers believe will commend itself so widely to builders and the public authorities that in due season it may transform the tenement-building system of the town. The plan was announced with Mrs. Vanderbilt's authority today by Dr. Henry L. Shively of the Vanderbilt clinic of the Presbyterian Hospital, and Architect Henry Atterbury Smith, who have the work under it in charge.

Dr. Shively has bought for Mrs. Vanderbilt eighteen city lots, extending from 77th to 78th streets between avenues A and B, with a frontage of 225 feet. On this plot four buildings are to be erected, to be known as the Shively sanitary tenements, after Mr. Smith's designs. They are to be built on a new pattern, although the several ideas that go to make it up have long been known. The novelty consists in the practical combination of them all in a form suited to give to sufferers from tuberculosis in their own homes the advantages of a sanatorium and to offer to healthy poor people the most healthful of living conditions in a big city. The buildings are to be made up of two, three, four and five-room apartments, offered at low rentals, and the four buildings are to house from 350 to 375 families. They are six stories high.

In presenting the plan, Dr. Shively remarked that Mrs. Vanderbilt had been not only interested in, but actively engaged in the work of the Vanderbilt clinic in tuberculosis cases for three or four years past, in the work of nursing the patients in their homes, supplying extra diet—for instance, milk and eggs—and in the removal of sufferers to sanitary quarters.

The fresh-air ward on the roof of the Presbyterian Hospital was given by her, and Dr. Shively said that it had been of incalculable use.

